# A practical framework for appropriate implementation and review of artificial intelligence (FAIR-AI) in healthcare

**DOI:** 10.1038/s41746-025-01900-y

**Published:** 2025-08-11

**Authors:** Brian J. Wells, Hieu M. Nguyen, Andrew McWilliams, Matt Pallini, Amy Bovi, Andrew Kuzma, Justin Kramer, Shih-Hsiung Chou, Timothy Hetherington, Patricia Corn, Yhenneko J. Taylor, Audrey Cuison, Mary Gagen, McKenzie Isreal, FAIR-AI Consortium, Brian J. Wells, Hieu M. Nguyen, Andrew McWilliams, Matt Pallini, Amy Bovi, Andrew Kuzma, Justin Kramer, Shih-Hsiung Chou, Timothy Hetherington, Patricia Corn, Yhenneko J. Taylor, Audrey Cuison, Mary Gagen, McKenzie Isreal, Oguz Akbilgic, Katie Barr, Alicia Bowers, Rikki Caffrey, Michael S. Carroll, Matthew CiRullo, Stephen M. Downs, Natalie Hardy, Jason Heuay, Kristina Katzovitz, Eric Kirkendall, Elsie Lindgren, Lindsey Lonergan, Elissa McKinley, Nicholas M. Pajewski, Laura Sak-Castellano, Erika Setliff, Gabe Wright

**Affiliations:** 1 Department of Biostatistics and Data Science, Wake Forest University School of Medicine, Winston-Salem, NC USA; 2 Center for Health System Sciences, Atrium Health, Charlotte, NC USA; 3 Department of Internal Medicine, Division of Hospital Medicine, Atrium Health, Charlotte, NC USA; 4 Advanced Analytics, Enterprise Data Science, Advocate Health, Charlotte, NC USA; 5 Conflict, Ethics, and Influence Program, Advocate Health, Milwaukee, WI USA; 6 Department of Family and Community Medicine, Wake Forest University School of Medicine, Winston-Salem, NC USA; 7 Compliance and Integrity, Advocate Health, Winston-Salem, NC USA; 8 Advanced Analytics, Advocate Health, Charlotte, NC USA; 9 Department of Cardiovascular Medicine, Wake Forest University School of Medicine, Winston-Salem, NC USA; 10 Advocate Health, Milwaukee, WI USA; 11 Innovation and Commercialization, Advocate Health, Charlotte, NC USA; 12 Clinical Ethics, Advocate Health, Chicago, IL USA; 13 Family Medicine, Atrium Health, Charlotte, NC USA; 14 Department of Pediatrics, Wake Forest University School of Medicine, Winston-Salem, NC USA; 15 Advocate Health, Oakbrook, IL USA; 16 Advocate Health, Winston-Salem, NC USA; 17 Patient Safety, Advocate Health, Milwaukee, WI USA; 18 Office of the General Counsel, Atrium Health, Charlotte, NC USA; 19 Cybersecurity Governance, Risk and Compliance, Advocate Health, Milwaukee, WI USA; 20 Audit Services and Enterprise Risk Management, Advocate Health, OakBrook, IL USA; 21 Virtual Critical Care (Southeast Region Critical Care), Atrium Health, Charlotte, NC USA

**Keywords:** Machine learning, Predictive medicine, Health services, Medical ethics, Health care, Information technology, Software

## Abstract

Health systems face the challenge of balancing innovation and safety to responsibly implement artificial intelligence (AI) solutions. The rapid proliferation, growing complexity, ethical considerations, and rising demand for these tools require timely and efficient processes for rigorous evaluation and ongoing monitoring. Current AI evaluation frameworks often lack the practical guidance for health systems to address these challenges. To fill this gap, we developed a prescriptive evaluation framework informed by a literature review, in-depth interviews with key stakeholders, including patients, and a multidisciplinary design workshop. The resulting framework provides health systems an outline of the resources, structures, criteria, and template documents to enable pre-implementation evaluation and post-implementation monitoring of AI solutions. Health systems will need to treat this or any alternative framework as a living document to maintain relevance and effectiveness as the AI landscape and regulations continue to evolve.

## Introduction

The healthcare industry is at an inflection point as the use of artificial intelligence-based tools rapidly expands, driven by the enhanced capabilities of modern electronic health record (EHR) systems and the advancement in artificial intelligence (AI) methods. The latest advancements in AI offer tremendous potential to improve patient outcomes, enhance patient experience, and increase efficiencies^1^. However, if hasty deployment of AI solutions bypasses rigorous evaluation steps, AI may paradoxically produce untoward results, such as introducing or amplifying health inequities, creating wasteful care, and causing harm to those intended to be helped^2^.

While AI has been used for clinical decision support in medicine for almost 50 years, evaluating the initial computer-based knowledge systems was relatively straightforward^3^. As AI use cases in healthcare expand, appropriately evaluating and monitoring AI solutions has become increasingly challenging due to more complex and, at times, inherently opaque AI models and methods with massive data requirements^4^. These challenges, combined with the rapid pace with which technology is being introduced and the increasing interest in utilizing innovative technologies, highlight the need for health systems to adopt new approaches for AI evaluation and governance. The approaches need to be consistent with the historically high standards healthcare has maintained for responsibly adopting new technology.

The necessity for oversight in healthcare is reflected in numerous publications demonstrating the gravity of potential risks that are uniquely present when AI intersects with decisions of consequence^5–7^. To harness the benefits of AI while appropriately managing its risks, health systems need to implement intentional, practical AI evaluation and governance strategies. Despite the recent hype and growing ubiquity of AI solutions, standardized approaches for guiding the pre-implementation review and post-implementation monitoring of AI in healthcare remain limited. Although the European Union (EU) AI Act is legally enforceable in Europe, it has drawn criticism for its lack of clarity and flexibility in defining “high-risk” AI—particularly in healthcare, where risk is highly context-dependent, varying by the specific tool, degree of human oversight, and clinical use case. In the United States, frameworks such as the Food and Drug Administration (FDA)’s Software as Medical Device (SaMD) guidance, National Institute of Standards and Technology (NIST)’s AI Risk Management Framework, and the AI Bill of Rights have emerged, but they are non-binding and provide limited practical guidance for implementation within real-world healthcare systems.

In the context of enterprise risk management, health systems seek to understand, quantify, and manage risk to all stakeholders, be that to patients, employees, or the organization. To effectively address the direct and indirect risks of implementing AI solutions, evaluation frameworks must be comprehensive, standardized, repeatable, and transparent. However, existing evaluation frameworks often fail to meet these criteria, as they tend to be overly theoretical, lack practical and actionable guidance, or focus too narrowly on specific aspects of risk^8–11^.

Considering these limitations, our organization, a large health system spanning the southeast and midwestern U.S., set out to create a practical, comprehensive AI framework focused on responsible AI implementation that can be applied in various healthcare settings. This project, Framework for the Appropriate Implementation and Review of AI (FAIR-AI) in healthcare, was guided by three aims: (1) to incorporate best practice recommendations from existing frameworks, guidelines, and regulations; (2) to understand the expectations and needs for an AI evaluation framework from a diverse set of health system stakeholders including patients, providers, operational leaders, and AI developers; and (3) to leverage a multidisciplinary group to synthesize best practice guidance and align stakeholder needs into a practical framework.

## Results

### Best practices and key considerations—narrative review

As a first step to inform the construct of FAIR-AI, we conducted a narrative review to identify the best practices and key considerations related to responsibly deploying AI in healthcare, these are summarized in Table 1. The results are organized into several themes including validation, usefulness, transparency, and equity.

**Table 1:** Best practices and key considerations in implementation of artificial intelligence

Theme	Best practices and key considerations
Validity	
	Choose appropriate metrics to assess model performance^12–14^.
	Evaluate whether the model achieves appropriate performance with consideration of the clinical context^14^.
	Conduct validation studies to assess the model’s applicability to real-world clinical practice^17,18^.
Usefulness	
	Assess the AI solution’s net benefit by weighing benefits and risks and considering workflows that mitigate risks^18,21–23^.
	Assess usefulness based on factors such as resource utilization, time savings, ease of use, workflow integration, end-user perception, alert characteristics (e.g., mode, timing, and targets), and unintended consequences^9,22,24^.
Transparency and equity	
	Disclose information about the data and methods used to create the AI system^25,26^.
	Disclose which patient characteristic variables that have historically been used to discriminate are included in the model and present clear justification^21,27–29^.
	Assess model performance across key patient subgroups^10,30,31^.
	Assess whether the AI system is equally accessible to those who may benefit^25^.
	Provide end-users with explanations and insights about the AI system’s processes and its potential biases and errors^32^.
	Notify patients when AI is being used and, when appropriate, to obtain their consent—particularly in sensitive or high-stakes situations^33,34^.

Numerous publications and guidelines such as TRIPOD and TRIPOD-AI have described the reporting necessary to properly evaluate a risk prediction model, regardless of the underlying statistical or machine learning method^12,13^. An important consideration in model validation is careful selection of performance metrics^14^. Beyond discrimination metrics like AUC, it is important to assess other aspects of model performance, such as calibration, and the F-score, which is particularly useful in settings with imbalanced data. For models that produce a continuous risk, probability decision thresholds can be adjusted to maximize classification measures such as positive predictive value (PPV) depending on the specific clinical scenario. Decision Curve Analysis can help evaluate the tradeoff between true positives and false positives to determine whether a model offers practical value at a given clinical threshold^15^. For regression problems, besides Mean Square Error (MSE), other metrics such as Mean Absolute Error (MAE) and Mean Absolute Percentage Error (MAPE) can also be examined^16^. It is important to establish a model’s real-world applicability through dedicated validation studies^17,18^. The strength of evidence supporting validation and minimum performance standards should align with the intended use case, its potential risks, and the likelihood of performance variability once deployed based on the analytic approach or data sources (Supplementary Fig 1)^14,17,18^. Applying these traditional standards to evaluate the validity of generative AI models is uniquely challenging and frequently not possible. While the literature in this area is nascent, evaluation should still be performed and may require qualitative metrics such as user feedback and expert reviews, which can provide insights into performance, risks, and usefulness^19,20^.

Deploying and maintaining AI solutions in healthcare requires significant resources and carries the potential for both risk and benefits, making it essential to evaluate whether a tool delivers actual usefulness, or a net benefit, to the organization, clinical team, and patients^21,22^. Decision analyses can quantify the expected value of medical decisions, but they often require detailed cost estimates and complex modeling. Formal net benefit calculations simplify this process by integrating the relative value of benefits versus harms into a single metric^18,23^. However, a lack of objective data, the specific context, or the nature of the solution may render these calculations impractical. In these cases, net benefit provides a construct to guide qualitative discussions among subject matter experts, helping to weigh benefits and risks while considering workflows that mitigate risks. Additionally, a thorough assessment of clinical utility may require an impact study to evaluate a solution’s effects on factors such as resource utilization, time savings, ease of use, workflow integration, end-user perception, alert characteristics (e.g., mode, timing, and targets), and unintended consequences^9,22,24^.

Given the potential for ethical and equity risks when deploying AI solutions in healthcare, transparency should be present to the degree that it is possible across all levels of the design, development, evaluation, and implementation of AI solutions to ensure fairness and accountability (https://nvlpubs.nist.gov/nistpubs/ai/nist.ai.100-1.pdf; http://data.europa.eu/eli/reg/2024/1689/oj)^25^^,^^26^. Specifically due to the potential for AI to perpetuate biases that could result in over- or under-treatment of certain populations, there must be a clear and defensible justification for including predictor variables that have historically been associated with discrimination, such as those outlined in the PROGRESS-Plus framework: place of residence, race/ethnicity/culture/language, occupation, gender/sex, religion, education, socioeconomic status, social capital, and personal characteristics linked to discrimination (e.g., age, disability, sexual orientation)^21,27–29^. This is particularly important when these variables may act as proxies for other, more meaningful determinants of health. It is equally important to evaluate for patterns of algorithmic bias by monitoring outcomes for discordance between patient subgroups, as well as ensuring equal access to the AI solution itself when applicable^10,25,30,31^. Once an AI solution is implemented, transparency for end-users becomes a critical element for building trust and confidence, as well as empowering users to play a role in vigilance for potential unintended consequences. To achieve this post-implementation transparency, end-users should have information readily available that explains an AI solution’s intended use, limitations, and potential risks (https://www.fda.gov/medical-devices/software-medical-device-samd/transparency-machine-learning-enabled-medical-devices-guiding-principles)^32^. Transparency is also critical from the patient’s perspective. There is an ethical imperative to notify patients when AI is being used and, when appropriate, to obtain their consent—particularly in sensitive or high-stakes situations^33,34^. This obligation is heightened when there is no human oversight, when the technology is experimental, or when the use of AI is not readily apparent. Failing to disclose the use of AI in such contexts may undermine patient autonomy and erode trust in the healthcare system. Generative AI presents unique challenges in terms of transparency. For example, deep learning relies on vast numbers of parameters drawn from increasingly large datasets and may be inherently unexplainable. When transparency is lacking there should be a greater emphasis on human oversight and education on limitations and risks, and this is an area of ongoing research^20^.

### Stakeholder needs and priorities—interviews

Several systematic reviews emphasize the importance of stakeholder engagement in the design and implementation of AI solutions in healthcare; however, this aspect is often overlooked in the existing frameworks^35,36^. To create a practical and useful framework for health systems, we borrowed from user-centric design principles to first assess stakeholders’ priorities for an AI framework and their criteria for evaluating its successful implementation. We interviewed stakeholders including health system leaders, AI developers, providers, and patients. Our findings were previously presented at the 17^th^ Annual Conference on the Science of Dissemination and Implementation, hosted by AcademyHealth^37^.

The stakeholders expressed multiple priorities for an AI framework, particularly the need for: (1) risk tolerance assessments to weigh the potential patient harms of an AI solution against expected benefits, (2) a human-in-the-loop of any medical decisions made using an AI solution, (3) consideration that available, rigorous evidence may be limited when reviewing new AI solutions, and (4) awareness that solutions may not have been developed on diverse patient populations or data similar to the population in which a use case is proposed. Interviewees also highlighted the importance of ensuring that AI solutions are matched to institutional priorities and conform to all relevant regulations. They noted regulations can pose unique challenges for large, multi-state health systems. While patient safety and outcomes were identified as paramount, stakeholders also detailed the need for an AI framework to evaluate the impact of potential solutions on health system employees.

When evaluating the successful implementation and utilization of an AI framework, stakeholders were consistent in explaining that the review process must operate in a timely manner, provide clear guidelines for AI developers, and ensure fair and consistent review processes that are applicable for both internally and externally developed solutions. Multiple interviewees cited the challenges presented by the rapid pace of AI innovation, expressing concerns that an overly bureaucratic and time-consuming review process could hinder the health system’s ability to keep pace with the wider healthcare market. Similarly, multiple senior leaders and AI developers explained that a successful AI framework would both encourage internal innovation and streamline the implementation of AI solutions in a safe manner.

### Framework for the appropriate implementation and review of AI (FAIR-AI) in healthcare

**Fig. 1:**
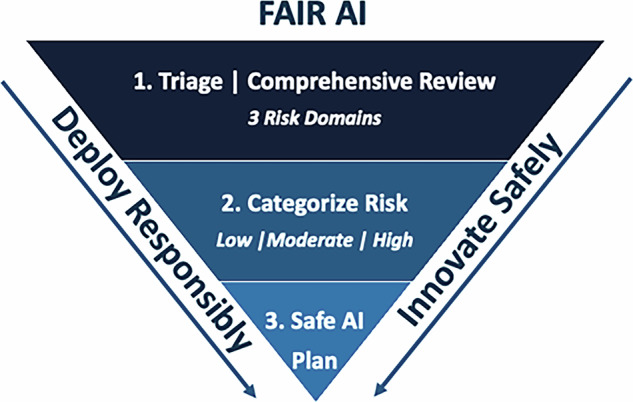
Core components of FAIR-AI. FAIR-AI aims towards safe innovation and responsible deployment with AI solutions in healthcare. This is achieved through a process centered around (1) comprehensive review organized by risk domains; (2) categorization of an AI solution into low, moderate, or high risk; and (3) Safe AI Plan consisting of monitoring and end-user transparency requirements.

Findings from stakeholder interviews informed our design workshop efforts, which included health system leaders and experts in AI, with workshop participants providing explicit guidance on how to best construct the FAIR-AI to meaningfully integrate stakeholder feedback. The project team leveraged design workshop activities and participant expertise to develop a set of requirements for health systems seeking to implement AI responsibly. FAIR-AI provides a detailed outline of: (i) foundational health system requirements—artifacts, personnel, processes, and tools; (ii) inclusion and exclusion criteria that specifically detail which AI solutions ought to be evaluated by FAIR-AI, thus defining scope and ensuring accountability; (iii) review questions in the form of a low-risk screening checklist and an in-depth review that provides a comprehensive evaluation of risk and benefits across the areas of development, validation, performance, ethics and equity, usefulness, compliance and regulations; (iv) discrete risk categories that map to the review criteria and are assigned to each AI solution and its intended use case; (v) safe implementation plans including monitoring and transparency requirements; (vi) an AI Label that consolidates information in an understandable format. These core components of FAIR-AI are also displayed in Fig. 1.

Implementing a responsible AI framework requires that health systems have certain foundational elements in place: (i) artifacts include a set of guiding principles for AI implementation and an AI ethics statement (examples are shown in Supplementary Table 1), both of which should be endorsed at the highest level of the organization; (ii) personnel including an individual (or a team) with data science training who are accountable for reviews; (iii) a process for escalation to an institutional decision-making body with the multidisciplinary expertise needed to assess ethical, legal, technical, operational, and clinical implications, with the authority to act; and (iv) an inventory tool that serves as a single source of truth catalog that enables accountability for review, monitoring, and transparency requirements. It is important to establish that the AI evaluation framework does not replace but rather supports existing governance structure. Additionally, while the overarching structure of an AI governance framework like FAIR-AI may remain consistent over time, the rapid pace of change in technology and regulations requires a process for regular review and updating by subject matter experts.

As the first step in FAIR-AI, an AI solution needs to go through an intake process. Individual leaders who are responsible for the deployment of AI solutions within the enterprise are designated as business owners; for clinical solutions, the business owner is a clinical leader. In this framework, we require the business owner of an AI solution to provide a set of descriptive items through an intake form including: (i) existing problem to solve; (ii) clearly outlined intended use case; (iii) expected benefits; (iv) risks including worst-case scenario(s); (v) published and unpublished information on development, validation, and performance; and (vi) FDA approvals, if applicable.

Next, we describe the inclusion and exclusion criteria for AI solutions to be applicable to FAIR-AI. Based on the premise that enterprise risk management must cast a wide net to be aware of potential risks, the inclusion for FAIR-AI review starts with a broad, general definition of AI solutions, which intentionally also includes solutions that do not directly relate to clinical care. We adopted the definition of AI from Matheny et al., as “computer system(s) capable of activities normally associated with human cognitive effort”^38^. We then provide additional scope specificity by excluding three general areas of AI. First, simple scoring systems and rules-based tools for which an end-user can reasonably be expected to evaluate and take responsibility for performance. Second, any physical medical device that also incorporates AI into its function, as there are well-established FDA regulations in place to evaluate and monitor risks associated with these devices (https://www.fda.gov/medical-devices/classify-your-medical-device/how-determine-if-your-product-medical-device). Third, any AI solution being considered under an Institutional Review Board (IRB)-approved research protocol that includes informed consent for the use of AI when human subjects are involved. Inclusion and exclusion criteria like these will need to be adapted to a health system’s local context.

Risk evaluation considers the magnitude and importance of adverse consequences from a decision; and in the case of FAIR-AI, the decision to implement a new AI solution^39^. As there are numerous approaches and nomenclatures to define risk, local consensus on a clear definition is a critical initial step for a health system. We aimed for simplicity in our risk definition and the number of risk categories to ensure interpretability by diverse stakeholders. Additionally, we opted to pursue a qualitative determination of risk and avoid a purely quantitative, composite risk score approach. The requisite data rarely exist to perform such risk calculations reliably, and composites of weighted scores have the potential to dilute important individual risk factors as well as the nuance of risk mitigation offered by the workflows surrounding AI solutions (for example, requiring a human review of AI output before an action is taken). Thus, FAIR-AI determines the magnitude and importance of potential adverse effects through consensus between subject matter experts from a data science team, the business leader requesting the AI solution, and ad hoc consultation when additional expertise is needed. In this exercise, the group leverages published data and expert opinion to outline hypothetical worst-case scenarios and the harms that could occur as an indirect or direct result of output from the proposed AI solution. The consensus determines if those harms are minor, or not minor; and if not minor, are they sufficiently mitigated by the related implementation workflow and monitoring plan. This risk framework is like that proposed by the International Medical Device Regulators Forum (https://www.imdrf.org/documents/software-medical-device-possible-framework-risk-categorization-and-corresponding-considerations). It is important here to note that every AI solution should be reviewed within the context of its intended use case, which includes the surrounding implementation workflows.

As prioritized by our stakeholders, a responsible AI framework should be nimble enough to allow quick but thorough reviews of AI solutions that have a low chance of causing any harm to an individual or the organization. To that end, FAIR-AI incorporates a 2-step process: an initial low-risk screening pathway and a subsequent in-depth review pathway for all solutions that do not pass through the low-risk screen. For an AI solution to be designated low-risk, it must pass all the low-risk screening questions (Table 2). Should answers to any of the screening questions suggest potential risks, the AI solution moves on to an in-depth review guided by the questions presented in Table 3. The in-depth review involves closer scrutiny of the AI solution by the data scientist and business owner and mandates a higher burden of proof that the potential benefits of the solution outweigh the potential risks identified during the screening process. If any of the in-depth review questions results in a determination of high risk, then the solution is considered high risk. It is also possible that the discussion between the data scientist and business owner will lead to a better understanding of the solution that results in a change to the answers to one or more of the low-risk screening, resulting in a low-risk designation.

**Table 2:** Low-risk screening questions

	Question	No or N/A	YES
1	Adverse effects Is there reasonable potential that the AI introduces adverse effects that may be more than minor for patients, employees, and/or individuals? • There should be adequate evidence of implementation in similar settings to properly determine the potential for minor adverse effects.	Low risk	Proceed to in-depth review
2	Trust Is there reasonable potential that the AI may negatively impact trust between provider (or health system) and patient(s)?	Low risk	High risk Proceed to in-depth review
3	AI features, equity screen Does the AI algorithm incorporate (or inappropriately exclude) characteristics^a^ that have historically been used to discriminate? • ‘YES’, if the developer cannot or will not show supporting evidence and clear supporting rationale.	Low risk	Proceed to in-depth review
4	AI output, equity screen Is it possible the AI will lead to decisions that differ across characteristics^a^ that have historically been used to discriminate? • ‘YES’ if the intended problem to solve is one where disparities exist |(e.g., access to healthcare resources, health outcomes, job applications, etc.).	Low risk	Proceed to in-depth review
5	Vulnerability considerations^b^ Does the AI implementation intersect with any of the following healthcare settings/functions/populations: • Beginning of life (pre, peri, neo-natal) • End of life (hospice, DNR/code status, palliative care, advance directives) • Consent for treatment/research • Capacity for decision making	Low risk	High risk Proceed to in-depth review
6	Decision support Is the solution intended to provide decision support for any of the following? a. Medical coding b. Medical billing c. Employment or human resources d. Diagnosis, treatment, or prevention of disease	Low risk	Proceed to in-depth review
7	Sensitive data Does the AI interface with data that may require special consideration? a. Recording individuals b. Facial recognition c. Fingerprints d. Genetic data e. Claims/payor data f. Other sensitive data	Low risk	Proceed to in-depth review
8	Output explainability Will it be difficult for the intended user to understand how the AI solution arrived at its output or recommendation?	Low risk	Proceed to in-depth review
9	Ease of monitoring Post implementation, does the AI solution require advanced expertise to adequately monitor for expected and unexpected risks and benefits? • A ‘NO’ answer indicates the risks, any adverse effects, and benefits must be able to be routinely tracked by the business owner.	Low risk	Proceed to in-depth review
10	Other concern(s) Does the reviewer have any other significant concerns about the AI not captured within the low-risk screen?	Low risk	High Risk Proceed to in-depth review

*AI* Artificial Intelligence, *N/A* not available.

^a^PROGRESS-Plus: place of residence, race/ethnicity/culture/language, occupation, gender/sex, religion, education, socioeconomic status, social capital, personal characteristics associated with discrimination^29^.

^b^Vulnerability: The conditions determined by physical, social, economic, and environmental factors or processes which increase the susceptibility of an individual, a community, assets, or systems to the impacts of hazards (World Health Organization).

**Table 3:** In-depth review questions

	Question	No	Yes	Uncertain
1^b^	1.1 Software as a medical device (SaMD) Has the FDA cleared or approved the AI as SaMD^a^? • The business owner is responsible for producing the FDA letter.	Continue to 1.2	Continue to 1.3	n/a
	1.2 SaMD Could the software meet the FDA definition of software as a device? a. The AI acquires, processes, or analyzes a medical image or signal related to a patient’s health. If this statement is TRUE, answer “YES”. b. The AI displays medical information about a patient, study or guideline. If this statement is TRUE, answer “NO”. c. The AI provides recommendations to a health care professional about prevention, diagnosis, or treatment of a disease AND provides the basis for recommendations, so the health care professional is not relying solely on the AI output for decision making. If this statement is TRUE, answer “NO”.	Moderate risk Continue to 2	High risk Continue to 2	High risk Continue to 2
	1.3 SaMD Is the FDA approval of the AI as SaMD for the intended use within the healthcare organization? • The vendor and/or business owner are responsible for providing the FDA confirmation letter and all supporting documentation or data to allow for this determination.	High risk	Moderate risk	n/a
2	Potential for significant adverse effects Potential adverse effects are notable and could have a significant negative impact on patients, teammates, individuals, or the enterprise? • There should be adequate evidence of implementation in other similar settings to support a ‘NO’ answer. • The business owner is responsible for identifying supporting documentation	Moderate risk	High risk	High risk
3	Adverse effects and workflows Potential adverse effects are not minor but are adequately addressed by workflows to mitigate/control the risk?	High risk	Moderate risk	High risk
4	Net benefit There is substantial evidence that supports the benefits outweigh the risks that are expected from AI implementation? • Evidence should include implementation in other similar settings to support a ‘YES’ answer. • The business owner is responsible for identifying supporting documentation.	High risk	Moderate risk	High risk
5	AI features, equity in depth If the AI uses features that include characteristics^c^ historically used to discriminate, then adequate evidence is provided for how they influence the output in the context of the intended use?	High risk	Moderate risk	High risk
6	AI output, equity in depth Adequate evidence is provided that the AI solution performs well in all key subgroups? • E.g., a model appropriately ranks patients according to risk and does not systematically underestimate or overestimate risk.	High risk	Moderate risk	High risk
7	Access, equity Is the AI system equally accessible to those who may benefit?	High risk	Moderate risk	High risk
8	Medical billing, coding, human resource a. Is an output of the AI that is related to medical billing or medical coding made part of a patient’s permanent record or released to a third party without the intervention of a human? b. Does the AI rank or categorize applicants or teammates for an intended use that is HR related?	Moderate risk	High risk	n/a
9	Privacy/transparency a. Does the AI solution record an individual without their knowledge? b. Is the organization ethically obligated to provide an explicit explanation that AI is being used or need to consent that AI is being used, but that is not part of solution or workflow?^d^ (e.g., based on potential risk(s) or if no human is in the loop) c. Does the AI solution analyze personal data that may lead to profiling or categorizing of individuals (excluding risk scoring for clinical diagnosis or clinical workflow prioritization)?	Moderate risk	High risk	n/a
10	Development and validity Transparent reporting of development and validation steps is available AND no concerns are identified when evaluated against contemporary published AI reporting standards. If this statement is TRUE, answer “YES”. • Answer ‘NO’, if supporting evidence is insufficient. • Answer ‘NO’, if concerns are present regarding the general validity of the model. • Answer ‘NO’ if the AI solution’s methods or outputs are “blackbox”^e^ and the AI implementation creates the potential for anything more than minor adverse effects on patients, employees, or individuals.	High risk	Moderate risk	n/a
11	External performance a. Substantive evidence of external performance exists to the level that a local validation is not required? OR b. The development and validation data/environment are expected to be so similar to the local data/environment that local confirmation is NOT necessary (e.g., radiology imaging)?	High risk	Moderate risk	n/a
12	Human oversight Is the AI solution directly or indirectly tied to workflow(s) that automate an action, documentation, or patient communication without human review, which may adversely affect patient health outcomes?	Moderate risk	High risk	n/a
Carry forward low-risk screening questions that are high risk				
13	Sensitive data Does the AI interface with data that may require special consideration? a. Recording individuals b. Facial recognition c. Fingerprints d. Genetic data e. Claims/payor data f. Other sensitive data	n/a	High risk	High risk
14	Trust Is there reasonable potential that the AI may negatively impact trust between provider (or health system) and patient(s)?	n/a	High risk	High risk
15	Vulnerability considerations^f^ Does the AI implementation intersect with any of the following healthcare settings/functions/populations: • Beginning of life (pre, peri, neo-natal) • End of life (hospice, DNR/code status, palliative care, advance directives) • Consent for treatment/research • Capacity for decision making	n/a	High risk	High risk
16	Other concern(s) Does the reviewer have any other significant concerns about the AI not captured within the low-risk screen?	n/a	High risk	High risk

*FDA* Food and Drug Administration, *AI* Artificial Intelligence, *n/a* not available.

^a^SaMD: Software as Medical Device (https://www.fda.gov/regulatory-information/search-fda-guidance-documents/clinical-decision-support-software).

^b^Question 1 uses language from FDA guidance to designate solutions as high risk if they appear to require FDA approval for their intended use but do not have it.

^c^PROGRESS-Plus: place of residence, race/ethnicity/culture/language, occupation, gender/sex, religion, education, socioeconomic status, social capital, personal characteristics associated with discrimination^29^.

^d^Workflow refers to implementations of AI where patient disclosure and consent are already integrated into the clinical process. This question is intended to highlight cases where such disclosure or consent is not already part of the established process, and where separate consideration may be ethically required.

^e^“Blackbox” could mean either the information is proprietary and not shared or a deep learning model, which due to its complexity cannot be understood.

^f^Vulnerability: The conditions determined by physical, social, economic, and environmental factors or processes which increase the susceptibility of an individual, a community, assets, or systems to the impacts of hazards (World Health Organization).

**Fig. 2:**
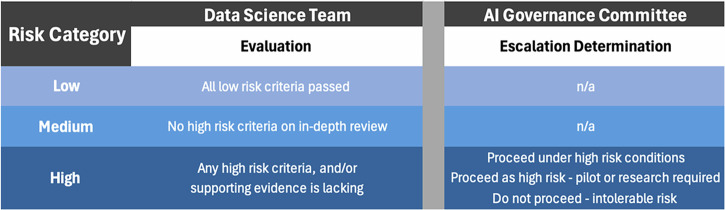
Risk categories as determined by FAIR-AI evaluation and escalation to AI Governance. This figure outlines the risk categorization process in FAIR-AI. The Data Science Team uses Low-risk Screening Questions and In-depth Review Questions to categorize the risk level of an AI solution. If given a high risk designation, the AI solution is escalated to the AI Governance Committee to determine whether deployment can proceed.

After the FAIR-AI review, which is described in detail in the next section, each AI solution is designated as low, moderate, or high risk according to the following definitions (Fig. 2):

- Low risk: Potential adverse effects are expected to be minor and should be apparent to the end-user and business owner. No ethical, equity, compliance, or regulatory concerns were identified during a low-risk screen.
- Moderate risk: Based on an in-depth review, one or more of the following are present: (1) potential adverse effects are not minor but are adequately addressed by workflows; (2) ethical, equity, compliance, or regulatory issues are suspected or present, but are appropriately mitigated.
- High risk: Based on an in-depth review, one or more of the following are present: (1) potential adverse effects are notable and could have a significant negative impact on patients, teammates, individuals, or the enterprise; (2) ethical, equity, compliance, or regulatory issues suspected or present, but not adequately addressed; (3) insufficient evidence exists to recommend proceeding with implementation.

For our health system, all AI solutions designated as high risk are escalated to the AI Governance committee where they undergo a multidisciplinary discussion. The discussion results in one of three final designations: (i) proceed to implementation under high-risk conditions; (ii) proceed to a pilot or research study; or (iii) do not proceed, implementation would create an intolerable risk for the organization.

The FAIR-AI framework is designed to encompass the full range of AI solutions in healthcare, including many that will not require in-depth review and can be designated low risk—such as those supporting back-office functions, cybersecurity, or administrative automation. Examples of moderate-risk AI tools in healthcare include solutions that support—but do not replace—clinical or administrative decision-making. These tools may influence patient care or documentation, but their outputs are generally explainable, subject to human review, and integrated into existing workflows that help mitigate risk. Examples of high-risk AI tools in healthcare include those that directly influence clinical care, diagnostics, or billing—particularly when used without consistent human oversight. They may also be deployed in sensitive contexts, such as end-of-life care or other high-stakes medical decisions. These tools often rely on complex, opaque models that can perpetuate bias, affect decision-making, and lead to significant downstream consequences if not rigorously validated and continuously monitored.

After application of the low-risk screening questions, the in-depth review questions (if necessary), and completion of the AI Governance committee review (if necessary), the proposed solution is assigned a final risk category, and a FAIR-AI Summary Statement is completed (an example is presented in Supplementary Box 1). At this point, an AI solution may need to go through other traditional governance requirements like a cyber security review, financial approvals, etc. If the AI solution ultimately is designated to move forward with implementation, then the data science team and business owners collaboratively develop a Safe AI Plan as outlined below.

**Fig. 3:**
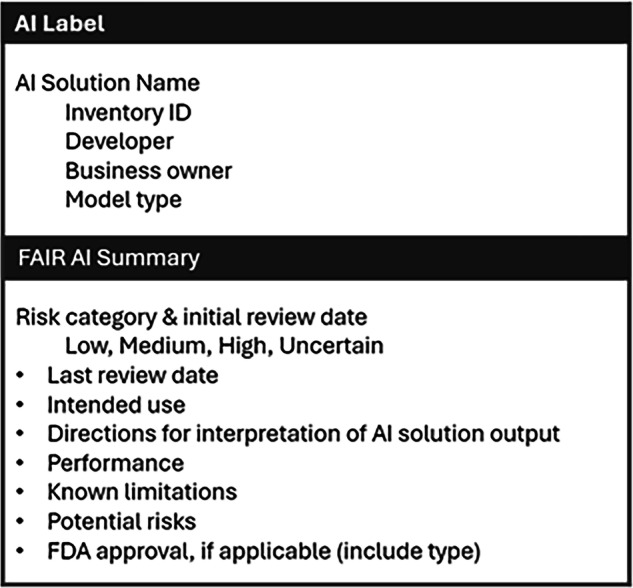
AI transparency label. As part of the Safe AI Plan, some AI solutions may be required to have an AI Label to provide end-user transparency. The AI Label includes an overview of the AI solution and a concise summary of the FAIR-AI review.

The first component of the Safe AI Plan concerns monitoring requirements. Implemented AI solutions need continuous monitoring as they may fail to adapt to new data or practice changes, which can lead to inaccurate results and increasing bias over time^40,41^. Similarly, when AI solutions are made readily available in workflows, it becomes easier for the solution to be used outside of its approved intended use case, which may change its inherent risk profile. For these reasons, FAIR-AI requires a monitoring plan for every deployed AI solution consisting of an attestation by the business owner at regular intervals. The attestation affirms that: (i) the deployment is still aligned with the approved use case; (ii) the underlying data and related workflows have not substantially changed; (iii) the AI solution is delivering the expected benefit(s); (iv) no unforeseen risks have been identified; and (v) there are no concerns noted related to new regulations. If the original FAIR-AI review identified specific risks, then the attestation also includes an approach to evaluate each risk along with metrics (if applicable). These evaluation metrics may range from repeating a standard model performance evaluation to obtaining periodic end-user feedback on accuracy (e.g., for a generative AI solution). The second component of the Safe AI Plan is transparency requirements. All solutions categorized as high risk also require an AI Label (Fig. 3) and end-user education at regular intervals. In situations where an end-user could potentially not be aware they are interacting with AI instead of a human, the business owner must also design implementation workflows that create transparency for the end-user (e.g., an alert, disclaimer, or consent as applicable).

## Discussion

Health systems are under growing pressure to adopt an increasingly wide array of AI solutions some of which have enormous potential to transform healthcare, but many also introduce complex potential risks. The FAIR-AI framework described in this paper offers a prescriptive, practical, and scalable approach for evaluating AI solutions for use in healthcare. We have distilled the approach into a concise set of questions that a data science team member can use to quickly triage AI solutions, triggering a more time-intensive, rigorous review only when necessary. For example, since the implementation of FAIR-AI within our health system, approximately 50% of the reviewed AI solutions have been triaged as low-risk. This practical approach is necessary given the volume of new solutions released and as AI becomes more ubiquitous across healthcare. By establishing formal review criteria and a consistent risk assessment process, institutions can ensure well-documented, defensible recommendations. Ultimately, by implementing FAIR-AI or a similar framework, health systems can foster a culture that upholds high standards for both internally and vendor-developed AI solutions, protecting patients and the care team, while being an early adopter harnessing actual AI benefits.

There are many challenges to implementing and maintaining the framework we have developed. Successful implementation requires support from institutional leadership, along with the allocation of resources to maintain documentation, manage new requests, and ensure proper monitoring. Team members tasked with screening requests must be empowered to reject requests for solutions that do not provide adequate documentation for a thorough review, otherwise, the process may become slow and inefficient as they search for information. In our early experience, we have found many AI solutions lack the evidence needed to support implementation and first require further research or pilot testing, which demands substantial resources from either the health system or the vendor. Generative AI solutions present significant challenges when they intersect with patient care, particularly around the difficulty in explaining how a tool functions, the opaque nature of the data used for training, the lack of standardized performance, the extensive manual effort required to review output, the need for infrastructure to obtain user-feedback, and mechanisms for reporting inaccuracies. An often overlooked but critical challenge to the responsible implementation of AI is the significant training required for both evaluators and end-users. Several recently published guidelines provide structured approaches for assessing the reliability and transparency of large language models in healthcare. We recognize the importance of these emerging frameworks and plan to expand our AI evaluation framework to incorporate relevant elements from them. However, integrating these considerations will take time, as adapting existing validation strategies for generative AI requires careful refinement to ensure a practical, efficient, and reproducible process that aligns with stakeholder needs^42,43^.

At our organization, we plan to review and adapt FAIR-AI at least annually, due to the rapid changes in the field and regulatory environment. For example, AI tools themselves are being used increasingly to monitor other AI solutions for safety, and future iterations of FAIR-AI will need to account for this evolving area. As AI solutions become pervasive across most workflows, all teammates play a role in being vigilant with an awareness of AI’s inherent limitations, security risks, and ethical considerations. To address this need to democratize responsibility, we are developing accompanying education that will enhance our organization’s responsible AI culture.

There are numerous limitations to our approach to evaluating AI solutions as described in this paper. Our evaluation and monitoring processes require a significant commitment of time and resources. Some health systems may choose to rely only on evaluations provided by other entities, which reduces the burden on the health system and speeds up the adoption of new AI tools; however, this may introduce inherent bias and conflicts of interest. For smaller healthcare systems, regional partnerships or strategic relationships will likely need to be considered as an alternate escalation pathway for high-risk solutions but is beyond the scope of this manuscript. Regardless, our framework can help smaller organizations inform a structured approach to weigh the risks and benefits of AI. While the screening and in-depth review questions provide a structured approach, they are not exhaustive, and the effectiveness of the framework depends on the diligence and expertise of the evaluators. Additionally, this framework will require that organizations make modifications to meet their needs and risk tolerance and to ensure alignment with local regulatory requirements. Modifications may also be needed to ensure the screening and in-depth review questions are clear and provide consistent risk determinations with different reviewers. Future qualitative evaluations can explore areas that may be unclear or leading to discrepancies between reviewers and thus needing further refinement.

FAIR-AI provides a practical template for health systems to adopt a process for the rigorous evaluation and monitoring of AI solutions. The prescriptive framework guided by explicit criteria is intentionally designed for health systems to use at the speed and scale required in real-world settings. This framework will enable institutions to carefully balance the desire to adopt innovative solutions while maintaining the highest standards for patient and care team safety.

## Methods

### Best practices and key considerations—narrative review

We conducted a narrative review to inform the development of our AI evaluation framework, opting for a pragmatic and expert-guided approach rather than a formal and focused scoping or systematic review. Given the existence of recent systematic reviews on this topic, our objective was not to comprehensively catalog or compare existing frameworks, but to synthesize insights most relevant to real-world implementation^35,36^. This approach allowed us to prioritize issues based on stakeholder input, domain knowledge, and practical relevance.

For the narrative review, we utilized Google Scholar as the primary search engine to locate pertinent published frameworks and papers. Search terms included: *framework, guideline, evaluation, monitoring, transparency, explainability, artificial intelligence, validation, informatics, clinical decision support, ethics, equity, regulatory, legal, usefulness, risks, benefits, implementation, deployment, predictive model, machine learning, clinical utility, health*. Additionally, we reviewed institutional guidelines from the European Union’s Artificial Intelligence Act, the National Institute of Standards and Technology (NIST), and the U.S. Food and Drug Administration (FDA) and conducted citation tracking to identify influential works.

### Stakeholder needs and priorities—interviews

From March to April 2024, we conducted semi-structured interviews with executive leadership (*N* = 3), senior risk, compliance, and legal leaders (*N* = 6), data developers (*N* = 4), providers (*N* = 5), and patients (*N* = 5) from across our health system. We utilized purposive sampling methods to ensure we obtained stakeholder feedback from the five user domains (e.g., executive leadership) that we felt would be most impacted by the implementation of an AI framework^44^. Interviewees were identified by members of the study team, with recruitment outreach occurring either via email (for health system teammates) or telephone (patients). All potential participants were provided with information on the scope of the project, with interviews being scheduled for those interested. An interview guide was collaboratively developed by the study team, which included physicians, faculty, and health system leaders with expertise in ethics, equity, data science, and care delivery (Supplementary Note 1). Each interview lasted approximately 30 minutes, was completed via telephone or videoconference, and was facilitated by a male member of the study team, who is a PhD-level sociologist (JK) and holds a faculty appointment. All participants provided verbal consent prior to interviews commencing. Interviews were audio recorded and transcribed verbatim, with ATLAS.ti software aiding data analysis efforts. Transcripts were analyzed using both inductive and deductive coding methodologies, with thematic analysis employed to identify and organize emergent themes in the data. Three members of the study team collaboratively developed the coding dictionary (BJW, JK, AM), with the qualitative lead (JK) independently coding all transcripts and bringing any questions back to the team for review. Participants did not assist the study team with transcription verification, data analysis, or interpretation of findings. We followed the Consolidated criteria for Reporting Qualitative research for sharing our findings (Supplementary Note 2).

### Expert consensus—design workshop

We convened a half-day, in-person workshop to synthesize the best practices identified from the literature review, the priorities outlined by stakeholders, and the consensus recommendations from a diverse team of subject matter experts. This workshop provided an opportunity to bring health system leaders and AI experts together to review study team findings and leverage their expertise to advance FAIR-AI development. While this workshop included ample discussion and a review of study team findings, it was not itself a data collection activity, rather it was focused on progressing the structure and development of the FAIR-AI. Workshop participants included individuals with expertise in legal affairs, regulatory compliance, cyber security, ethics, clinical care, clinical informatics, data science, and research (*N* = 33). As the starting point for the workshop, the primary project team created a draft framework outline. This outline, along with background information, pertinent literature, and summaries of stakeholder needs, were shared with attendees for review prior to the meeting.

## Supplementary information

Supplementary information

## Data Availability

The datasets used and/or analyzed during the current study are available from the corresponding author upon reasonable request. Interview data are not made publicly available to protect the confidentiality of the interviewees, including senior leader participants.
