# Supplementary material for: A practical framework for appropriate implementation and review of artificial intelligence (FAIR-AI) in healthcare
Source: NPJ Digit Med. 2025 Aug 11;8:514. doi: 10.1038/s41746-025-01900-y (PMC12340025; doi:10.1038/s41746-025-01900-y)
Supplement: Supplementary file 1 — Supplementary information [file 41746_2025_1900_MOESM1_ESM.pdf]

A Practical Framework for Appropriate Implementation and Review of Artificial Intelligence (FAIR-AI) in Healthcare

Supplementary Information

Supplementary Figure 1. Strength of validation

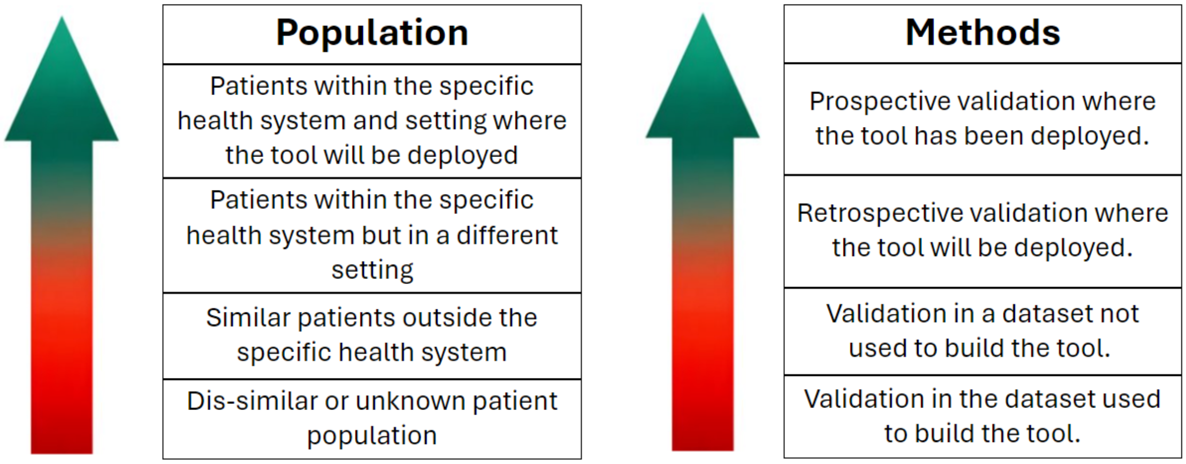

Validation requirements will depend on the specific tool, use-case, stakes, and workflow. Hi-stakes situations with tools that will have direct impacts on treatment decisions will require the greatest strength.

Supplementary Table 1. Examples – Guiding principles and Ethics Statement

**Ethics Statement**	
Our evaluation processes are designed to promote the responsible and ethical use of artificial intelligence (AI) in our healthcare system, ensuring that all AI tools we evaluate align with the highest standards of fairness, accountability, transparency, and responsibility. We are committed to ensuring that the deployment of AI respects the dignity, rights, and well-being of all individuals impacted by its use.	
**Guiding principles**	
• Equitable and ethical	It is our collective responsibility to identify and mitigate potential bias, injustice, and harm from the use of AI solutions.
• Valid and reliable	AI solutions will be internally evaluated before implementation and periodically while in production to ensure continued valid and consistent results within the specified scope of use. The extent of the evaluation is commensurate with the type of AI and the potential risk and/or benefit.
• Transparent	Detailed, understandable explanations of performance and impact will be available for stakeholders. End users will be made aware when they are interacting with an AI model instead of a human. Levels of explanations and awareness are commensurate with the type of AI and the potential risk and/or benefit.
• Impactful	AI solutions will be selectively applied to defined organizational problems with attention to the workflows that support implementation and adoption.

Supplementary Box 1. Example – FAIR-AI Summary Statement

FAIR-AI Review Summary

FAIR-AI review for [solution name] was completed on [Month dd, yyyy] (ID#[id num]; developed by [vendor]).

[This review was escalated to [organization name]'s AI Governance (AIG) committee and] The AI application has been categorized as MODERATE RISK based on the Framework for Appropriate Implementation & Review of AI (FAIR-AI). This determination only applies to the AI review of the intended use case. Additional enterprise approvals may be required prior to implementation.

<u>Brief Description of AI Application</u>

- What is the solution?
- What type of AI/model does it use?
- What output does it produce?
- How often does it produce results or what triggers a result?

<u>Intended Use Case</u>

- Who will be using it?
- How will it be used?
- What is the desired outcome or benefit from incorporating this tool?

<u>Risk Determination</u>

The following FAIR-AI criteria were identified as risks during review, leading to a Moderate risk determination

- [Identified FAIR-AI Risk Category: Describe risk]
- [Add a bullet for each risk criteria that triggered moderate risk. The risk criteria listed should correspond to language in the FAIR-AI screening. Describe why it was triggered and what specific risk or negative outcome we are exposed to as a result.]

<u>Other Considerations</u>

[OPTIONAL SECTION. Add bullets for any other considerations or recommendations.]
[If this was escalated to AIG, describe why it was escalated and why it was lowered to Moderate]

<u>Safe AI Monitoring</u>

Once implemented, AI solutions may develop inaccuracies or bias over time due to changes in data, workflow, or use. To mitigate that risk, the business owner acknowledges the following Safe AI responsibilities for Moderate Risk AI Solutions.

Complete the Safe AI Attestation form every <#> months from implementation.
In partnership with Enterprise Data Science, create a Safe AI Metric plan (including metric, methodology, frequency, and next due date) for each identified risk and provide updates during the Safe AI Attestation, as required.
Notify Enterprise Data Science of any changes to the AI solution or its use case.

Supplementary Note 1. Interview guide for the stakeholder interviews

Interview Introduction:
Participation in this interview is voluntary, you have the right to stop participating at any time, and you are able skip any questions that you wish. Choosing not to participate in this interview will not affect - [Insert applicable statement below]

- ***Advocate Teammates:*** *your employment at Advocate Health in any way.*
- ***Advocate Patients:*** *the care you receive at Advocate Health in any way.*

Interviews will be audio recorded and transcribed verbatim. Once transcripts are received and verified for accuracy, audio recordings will be deleted.

Interview transcripts will be de-identified, with your name removed from any resulting text files. We will not use your name in any publication or presentation from this work and we will do our best to protect your identity when sharing findings from this project.

- ***Advocate Teammates:*** *Although we will de-identify all interview data, given our small sample size and the information that we will discuss about your role within Advocate Health, it is possible that your identity may be inferred.*

Advocate Patients: *You will be provided with a $50 prepaid debit card as compensation for your time, which will be mailed to you at the completion of this interview.*

Interviews will focus on your thoughts pertaining to the use of Artificial Intelligence, or AI, by health systems and by health care providers. We are interested in learning about what you'd like to know about how health systems may use AI, where you would want to get this information from, and if you have any general concerns about health systems using AI. Finally, specific to your role as a ___________ [(n) executive leader, risk/compliance leader, data developer/scientist, provider, patient] at Advocate Health, we want to know about the things that you think Advocate Health should do to make sure that AI is used in a safe, ethical, and responsible manner within the health system.

Your feedback will help us as we work to develop a framework guiding Advocate Health's use of AI across the health system.

Content Introduction:
Advocate Health is working to define how AI is used safely within our healthcare system. AI has tremendous potential to transform healthcare, but also has real risks like providing false information and biased recommendations. We are interviewing people across our health system to understand different views on how we should approach AI responsibly. The term AI, or Artificial Intelligence, will refer to when computers perform activities that normally would be done by humans. AI can be used in many areas of healthcare, for example it may be used to analyze health data to predict health risk or make a diagnosis, help doctors see abnormal findings on x-rays, or create education for patients.

I. <u>General perceptions of AI in healthcare</u>

1. What are your general thoughts about health systems and health care providers using AI when providing care to patients?
    - How do you think that AI should be used in health care at Advocate Health? Why?

2. What do you think are some of the risks associated with using AI in healthcare?

3. What do you think are some of the benefits associated with using AI in healthcare?

4. Do you have any concerns about health systems and health care providers using AI?
    - When you think about those concerns, do you have ideas for how a health system might address them?

5. Before a new AI solution is used to help with patient care, what should a health system do to create trust among patients and/or providers?
    - Are there specific things that you would want to know about the new AI solution that could help you evaluate whether it is trustworthy? (e.g., is it safe?)

6. What does the ethical use of AI look like for a health system or for a health care provider?

7. How can Advocate Health show that we have carefully considered the ethical issues associated with our use of AI?
    - What information would you want to have to feel satisfied that we have thoroughly considered the ethical issues associated with our use of AI?
    - Is there anything else that you would want to know about how we regulate and use AI to evaluate if we are using it in a safe, ethical, and responsible way?
    - If you wanted to review information about Advocate Health's use of AI, where would you expect to find this information?

8. What equity issues are important to consider prior to a health system implementing a new AI solution?
    - What concerns would you have if an AI solution considered patient factors such as race, ethnicity, age, and/or gender?
    - How can health systems and health care providers prevent bias and/or discrimination in their use of AI?

II. <u>**Participant role and user persona specific questions.**</u>

Section Introduction: *The final few questions will focus on your specific role within Advocate Health and how AI might directly impact you.*

<u>Executive Leaders</u>

1. What are your expectations for an AI framework at Advocate Health?
    - What would the successful implementation and use of an AI framework look like?

2. What does a successful balance between innovation and safety look like for AI within Advocate Health?

<u>Government, Risk, Legal, and Compliance Leaders</u>

1. Given your role, what would you need from a framework that governs the use of AI across Advocate Health?
    - Are there any specific things that you think are essential for the AI framework to address?

2. What does a successful balance between innovation and safety look like for AI within Advocate Health?

<u>Data Scientists and Developers</u>

1. For developers wanting to incorporate AI into their projects and interventions, how would an enterprise-wide framework governing the use of AI at Advocate Health impact that work?
    - What are the key elements that developers would need to know from the AI framework to ensure development work ultimately leads to implementation?

2. What does a successful balance between innovation and safety look like for AI within Advocate Health?

<u>Provider and Care Team End Users</u>

1. If AI is being used to help in a care decision, to what degree do you want to be informed that AI was involved?
    - Would your opinion change if there was not a human who had reviewed the recommendation first?
    - Would your opinion change if AI was involved in a lower-risk decision, like suggesting the best cough medicine, versus AI suggesting a serious diagnosis like cancer.
    - If you want to know that AI is involved in the care process, what information would you need to know?

<u>Patient and Community end Users</u>

1. If AI is being used to help in a care decision, to what degree do you want to be informed that AI was involved?
    - Would your opinion change if there was not a human who had reviewed the recommendation first?
    - Would your opinion change if AI was involved in a lower-risk decision, like suggesting the best cough medicine, versus AI suggesting a serious diagnosis like cancer.
    - If you want to know AI is involved in the care process, what information would you need to know?

Supplementary Note 2. COREQ (COnsolidated criteria for REporting Qualitative research) Checklist prepared for the stakeholder interviews

A checklist of items that should be included in reports of qualitative research. You must report the page number in your manuscript where you consider each of the items listed in this checklist. If you have not included this information, either revise your manuscript accordingly before submitting or note N/A.

Topic	Item No.	Guide Questions/Description	Reported on Page No.
**Domain 1: Research team and reflexivity**			
Personal characteristics			
Interviewer/facilitator	1	Which author/s conducted the interview or focus group?	12
Credentials	2	What were the researcher's credentials? E.g. PhD, MD	12
Occupation	3	What was their occupation at the time of the study?	12
Gender	4	Was the researcher male or female?	12
Experience and training	5	What experience or training did the researcher have?	12
Relationship with participants			12
Relationship established	6	Was a relationship established prior to study commencement?	12
Participant knowledge of the interviewer	7	What did the participants know about the researcher? e.g. personal goals, reasons for doing the research	12
Interviewer characteristics	8	What characteristics were reported about the inter viewer/facilitator? e.g. Bias, assumptions, reasons and interests in the research topic	12
**Domain 2: Study design**			
Theoretical framework			
Methodological orientation and Theory	9	What methodological orientation was stated to underpin the study? e.g.<br>grounded theory, discourse analysis, ethnography, phenomenology, content analysis	12
Participant selection			12
Sampling	10	How were participants selected? e.g. purposive, convenience, consecutive, snowball	12
Method of approach	11	How were participants approached? e.g. face-to-face, telephone, mail, email	12
Sample size	12	How many participants were in the study?	12
Non-participation	13	How many people refused to participate or dropped out? Reasons?	N/A
Setting			
Setting of data collection	14	Where was the data collected? e.g. home, clinic, workplace	12
Presence of nonparticipants	15	Was anyone else present besides the participants and researchers?	12
Description of sample	16	What are the important characteristics of the sample? e.g. demographic data, date	12

Data collection			12
Interview guide	17	Were questions, prompts, guides provided by the authors? Was it pilot tested?	12
Repeat interviews	18	Were repeat inter views carried out? If yes, how many?	12
Audio/visual recording	19	Did the research use audio or visual recording to collect the data?	12
Field notes	20	Were field notes made during and/or after the inter view or focus group?	12
Duration	21	What was the duration of the interviews or focus group?	12
Data saturation	22	Was data saturation discussed?	N/A
Transcripts returned	23	Were transcripts returned to participants for comment and/or correction?	12
**Topic**	**Item No.**	**Guide Questions/Description**	**Reported on Page No.**
**Domain 3: analysis and findings**			
Data analysis			
Number of data coders	24	How many data coders coded the data?	12
Description of the coding tree	25	Did authors provide a description of the coding tree?	N/A
Derivation of themes	26	Were themes identified in advance or derived from the data?	12
Software	27	What software, if applicable, was used to manage the data?	12
Participant checking	28	Did participants provide feedback on the findings?	12
Reporting			
Quotations presented	29	Were participant quotations presented to illustrate the themes/findings?<br>Was each quotation identified? e.g. participant number	N/A
Data and findings consistent	30	Was there consistency between the data presented and the findings?	N/A
Clarity of major themes	31	Were major themes clearly presented in the findings?	6
Clarity of minor themes	32	Is there a description of diverse cases or discussion of minor themes?	6

Developed from: Tong A, Sainsbury P, Craig J. Consolidated criteria for reporting qualitative research (COREQ): a 32-item checklist for interviews and focus groups. *International Journal for Quality in Health Care*. 2007. Volume 19, Number 6: pp. 349 – 357
